# Characterizing the genetic diversity and population structure of *Plasmodium knowlesi* in Aceh Province, Indonesia

**DOI:** 10.1371/journal.pone.0318608

**Published:** 2025-03-11

**Authors:** Pinkan P. Kariodimedjo, Nadia Fadila, Sydney R. Fine, Hidayat Trimarsanto, Chris Cotter, Leily Trianty, Michelle S. Hsiang, Jennifer Smith, Adam Bennett, Rintis Noviyanti, Farah N. Coutrier

**Affiliations:** 1 Malaria Pathogenesis Unit, Eijkman Institute for Molecular Biology, Jakarta Pusat, Daerah Khusus Jakarta, Indonesia; 2 Exeins Health Initiative, Jakarta Pusat, Daerah Khusus Jakarta, Indonesia; 3 Malaria Elimination Initiative, Institute for Global Health Sciences, University of California San Francisco, California, United States of America; 4 Eijkman Research Center for Molecular Biology, National Agency for Research and Innovation (BRIN), Cibinong, West Java, Indonesia; 5 Department of Women’s and Children’s Health, Uppsala University, Uppsala, Uppland, Sweden; 6 Department of Pediatrics, University of California San Francisco, Benioff Children’s Hospital, San Francisco, California, United States of America; 7 Department of Pediatrics, University of Texas Southwestern, Dallas, Texas, United States of America; 8 Department of Epidemiology and Biostatistics, University of California San Francisco, San Francisco, California, United States of America; 9 PATH, Seattle, Washington, United States of America; ICMR Regional Medical Research Centre Dibrugarh, INDIA

## Abstract

As in other parts of Southeast Asia, efforts to achieve or sustain malaria elimination in Indonesia have been threatened by the emergence of human infection with the primate species *P. knowlesi.* To understand the transmission dynamics of this species, investigation of *P. knowlesi* genetic diversity and population structure is needed. A molecular surveillance study was conducted in two phases between June 2014 and September 2018 at five primary health facilities in Aceh Province, Indonesia, an area nearing malaria elimination. Dried blood spot samples were collected from patients presenting with suspected malaria and testing positive for malaria by microscopy. PCR was performed for molecular confirmation and species identification. Forty-six samples were confirmed to be *P. knowlesi*, of which 41 were amplified with genotyping targeting ten known *P. knowlesi* microsatellite markers. For samples within a site, nearly all (9 of 10 loci) or all loci were polymorphic. Across sites, multiple identical haplotypes were observed, though linkage distribution in the population was low (index of association (I_A_^S^) = 0.008). The parasite population was indicative of low diversity (expected heterozygosity [HE] =  0.63) and low complexity demonstrated by 92.7% monoclonal infections, a mean multiplicity of infection of 1.06, and a mean within-host infection fixation index (F_ST_) of 0.05. Principal coordinate and neighbour-joining tree analyses indicated that *P. knowlesi* strains from Aceh were distinct from those reported in Malaysia. In a near-elimination setting in Indonesia, we demonstrate the first evidence that *P. knowlesi* strains were minimally diverse and were genetically distinct from Malaysian strains, suggesting highly localized transmission and limited connectivity to Malaysia. Ongoing genetic surveillance of *P. knowlesi* in Indonesia can inform tracking and planning of malaria control and elimination efforts.

## Introduction

Similar to surrounding countries in Southeast Asia, malaria remains a critical public health concern in Indonesia. Indonesia experienced the second largest burden of malaria cases in the Southeast Asia Region in 2021, second only to India. Nevertheless, between 2015 and 2021, malaria incidence was reduced by 26% and the malaria mortality rate was reduced by 19% in Indonesia [1]. Malaria transmission in Indonesia is highly heterogenous, although areas in eastern Indonesia and Indonesian Borneo to the north have relatively intense transmission [2–4]. There are 372 provinces that have eliminated malaria, 83 that are considered low (<1 case/1000 population) transmission, and many others that have mid (1–5 cases/1000 population), high (>5 cases/1000 population), or unknown levels of transmission [5].

Historically, most malaria infections in Indonesia have been caused by *Plasmodium falciparum* or *Plasmodium vivax* [6,7]. However, a non-human primate species, *Plasmodium knowlesi* was recently detected in Indonesia. The first documented cases of *P. knowlesi* in Indonesia were identified in Sumatra, the western part of Indonesia, in 2014 [8–10]. Additionally, the mosquito vectors that carry *P. knowlesi* are common in the same region [11]. *Macaca fascicularis* and *Macaca nemestrina*, the long-tailed and pig-tailed monkeys, are the natural hosts of *P. knowlesi* and can withstand acute (natural and research-induced) infections and live normally with low-level parasitaemia [12,13]. These *Macaca* species inhabit almost all regions of Aceh Province [14–16]. The challenge in identifying this species earlier was largely due to errors in species identification by microscopy, and limited sensitivity, or cross-reactivity of species, with some molecular detection methods [17].

Understanding the genetic epidemiology of *Plasmodium* in an endemic area can inform control and elimination efforts [18,19]. Other groups have published data on the genetic diversity of *P. falciparum* [20] and *P. vivax* [20,21] in Indonesia. Evidence from these studies suggests that there is risk for distinct parasite subpopulations to persist within malaria-endemic communities, which may be below the limit of detection using standard diagnostics, such as microscopy and rapid diagnostic tests. Furthermore, forest workers and other migrant workers with patterns of mobility between endemic and non-endemic areas contribute to sustaining malaria transmission [22,23]. Particularly in areas of low transmission, understanding the genetic diversity and population structure of parasites can help to characterize transmission dynamics [24].

There is a limited understanding of the transmission dynamics of *P. knowlesi*, which has recently emerged as the dominant malaria species in Aceh Province, Indonesia. Increased awareness and knowledge of local patterns of malaria transmission and parasite relatedness can facilitate targeting of transmission reservoirs. To address this gap in evidence, this study aimed to characterize the genetic diversity and population structure of *P. knowlesi* using microsatellite genotyping.

## Materials and methods

### Study sites and sample collection

The study was conducted in Aceh Province, Indonesia, an area that has experienced a dramatic decline in malaria cases due to strengthened control and elimination efforts following the 2004 Indian ocean tsunami. The original data for this retrospective study were collected in two phases: Phase 1 (June 2014 to December 2015) [8,17] and Phase 2 surveillance studies (April 2017 – March 2018) [25]. The archive samples were accessed on 30 September 2019. The authors did not have access to information that could identify individual participants during or after data collection. Identifying details of each patient were removed by proper anonymization.

Patients who presented at a health facility with suspected malaria were invited to participate in the study. Written informed consent was obtained for venous blood collection before malaria treatment. Blood draw volume ranged between 250 uL-3 mL based on the age of the patient.

**Fig 1:**
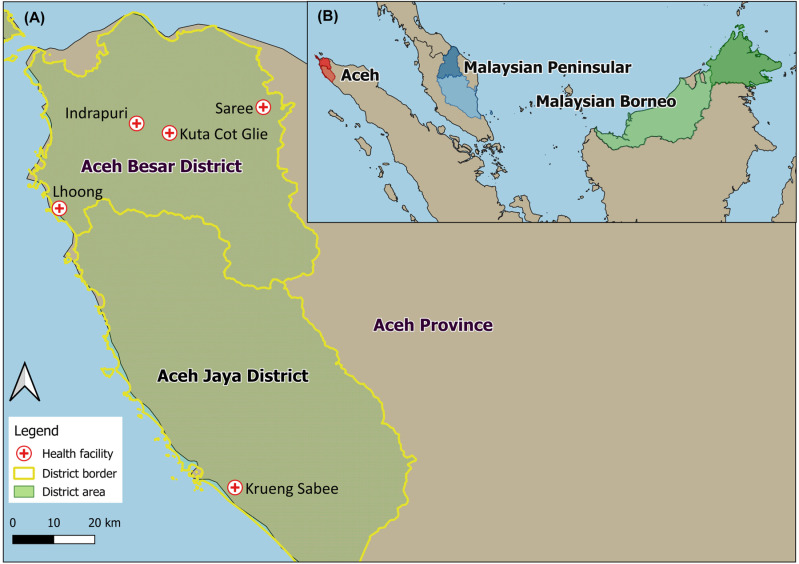
Maps of study sites and surrounding geographies. (A) Geographical locations of the five health facilities in this study (red plus sign). (B) Three geographic regions with established *P. knowlesi* transmission and with genetic data used in this study; Aceh (red), Peninsular Malaysia (blue), and Malaysian Borneo (green). (Source: Natural Earth, 2024).

The overall Annual Parasite Incidence (API) in Indonesia between 2014–2018 was 0.55 [26]. In Phase 1, primary facilities with the highest number of malaria cases in Aceh Besar District were included, which represented 78% of all cases reported from the district in 2013. Although most districts in Aceh have reached elimination, in 2022, Aceh Jaya District had 32 cases with an API =  0.33 [5]. In Phase 2, a facility from neighbouring Aceh Jaya District was added due to its high burden *P. knowlesi* (Fig 1).

Blood was collected to generate thick and thin blood smears for immediate diagnosis, to generate dried blood spots (DBS), and for collection in a heparin or EDTA tube for future analysis. Blood smears were fixed and stained with 3% Giemsa and read per national guidelines by a microscopist at the health facility. A thin smear was considered negative if no parasites were observed in 100 high-power fields. If parasites were identified, an additional 100 high-powered fields were examined to determine species. An expert level-certified microscopist performed quality assurance at the Banda Aceh Provincial Hospital for all positive malaria cases and 10% of randomly selected negatives. Results from the health facilities were confirmed by microscopists at the District and Provincial Health Offices.

### DNA extraction and molecular identification

DNA extraction from the blood samples was performed at the Eijkman Institute, Jakarta, using the QIAamp DNA Mini Kit (Qiagen, Germany). Species identification for *P. falciparum, P. vivax, P. malariae,* and/or *P. ovale* was performed using nested PCR targeting the18S rRNA gene [27] and identification of *P. knowlesi* was performed using the PCR targeting ssrRNA [28]. PCR products were visualized by gel electrophoresis on 1.5% agarose gel. Positive *P. knowlesi* samples then underwent microsatellite genotyping.

### Microsatellite genotyping of *P. knowlesi*

Microsatellite genotyping assays were conducted based on the ten trinucleotide markers (NC03_2, CD11_157, NC12_4, NC12_2, CD13_61, NC10_1, CD13_107, CD05_06, NC09_1) specific to *P. knowlesi* as previously described by Divis et al. [29]. Unique fluorescent dyes were assigned to each set of PCR primers to distinguish PCR products. Capillary electrophoresis was performed on an ABI 3100 Genetic Analyzer (Applied Biosystems, USA) using Genescan 600 LIZ as the molecular size standard (Applied Biosystems, USA). Genotyping analysis was conducted using GeneMapper Version 4.0 software (Applied Biosystems, USA) to identify genotypes. Electropherogram peaks were analyzed visually. To confirm genotyping quality, only allele peaks with a minimum height of 100 rfu were selected for analysis. The predominant allele plus any additional alleles with ≥ 33% of the height of the predominant allele were included in further analysis.

### Data analysis

A polyclonal infection was defined as the presence of multiple alleles at one or more loci. Multiplicity of Infection (MOI) was defined as the maximum number of alleles at any given locus and is a proxy for the number of genotypes per infection. All the observed alleles were used to measure polyclonality and MOI; however, only the predominant allele—or alleles—at each locus was used for the population structure analysis. The expected heterozygosity (H_E_), including factors of population diversity, and fixation index (F_ST_), is a measure of the distance between populations and was calculated using Arlequin software version 3.5. Pairwise identical alleles of each isolate were determined using GenAlEx version 6.5 within Microsoft Excel. A website-based software, LIAN (http://guanine.evolbio.mpg.de/cgi-bin/lian/lian.cgi.pl), was used to define the index of association (I_A_^S^), which is a measure of multilocus linkage disequilibrium (LD). Neighbour-joining tree and principal coordinate analysis (PCoA) plots (genetic distance matrix) were constructed by the ape package in R version 3.6.3.

We conducted cluster identification by Bayesian analysis using STRUCTURE software (Pritchard Lab, Stanford University) with parameters set according to Divis et al. [29]. The most probable *K*-value was determined based on the highest Δ*K* value generated by Evanno’s method through STRUCTURE harvester (https://alumni.soe.ucsc.edu/~dearl/software/structureHarvester/). STRUCTURE harvester generated an output of cluster assignment indices for visualization of clusters through CLUMPP software version 1.1.2 (Rosenberg Lab, Stanford University) and Distruct software version 1.1 (Rosenberg Lab, Stanford University).

### Ethical approval

Written informed consent was obtained from all adults or a parent/guardian for participants less than 18 years of age. This study was reviewed and approved by the Committee on Health Research Ethics of the National Institute of Health, Research and Development, Ministry of Health Republic of Indonesia (LB.02.01/5.2/KE.111/2014, LB.02.01/5.2/KE.211/2015, LB.02.01/5.2/KE.265/2016, LB.02.01/2/KE.083/2017), and the Committee on Human Research at the University of California, San Francisco (16-20220/171001, 16-20220/201201, 16-20220/232405, 16-20220/260683, 16-20220/289416, 13-10988/300323, 16-20220/337919). All the ethical approvals cover both the original studies and this retrospective study.

## Results and discussion

Malaria speciation was conducted from which a total of 6 samples tested positive for *P. falciparum* and 29 samples tested positive for *P. vivax*. Forty-six samples were confirmed to be *P. knowlesi,* and 41 of these successfully amplified at all ten loci targeted by microsatellite genotyping (Table 1). Isolates were analyzed for marker properties, genetic diversity, and population structure. To facilitate group comparisons and due to their physical proximity, isolates from Kuta Cot Glie (n = 1) and Indrapuri (n = 1) were combined as a single group. With the exception of a few samples (n = 3) that presented multiple peaks, most samples had a single peak representing a single genotype (MOI =  1.064). Polyclonal infections were observed from Krueng Sabee (n = 1) and Lhoong (n = 2). The genetic diversity of the isolates obtained was considered moderate, with an overall H_E_ =  0.634. Krueng Sabee isolates exhibited the highest genetic diversity (H_E_ =  0.7).

**Table 1:** Genetic diversity of *P. knowlesi* infections collected from five primary health centers in Aceh.

Region	Samples (n)	% of Polyclonal Infections (n/total)	MOI mean (range)	HE, mean ± SE
Krueng Sabee	5	20 (1/5)	1.2 (1-2)	0.700 ± 0.194
Lhoong	17	11.7 (2/17)	1.12 (1-2)	0.539 ± 0.251
Saree	14	0 (0/14)	1	0.637 ± 0.203
Kuta Cot Glie- Indrapuri	5	0 (0/5)	1	0.660 ± 0.280
Total	41	7.3 (3/41)	1.064	0.634 ± 0.232

### Genetic diversity and structure

**Fig 2:**
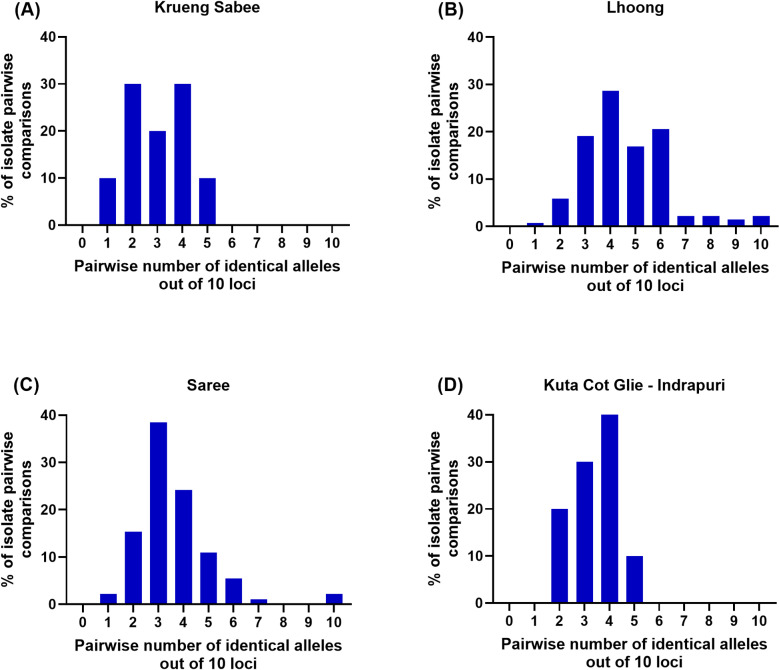
Pairwise comparisons of *P. knowlesi* isolates. These plots demonstrate the percentage of *P. knowlesi* isolate pairwise comparisons of 10 loci from (A) Krueng Sabee, (B) Lhoong, (C) Saree, and (D) Kuta Cot Glie–Indrapuri.

The diversity and genotyping for *P. knowlesi* microsatellite markers are summarised in S1 Table. Study isolates were characterized based on presence of a set of known *P. knowlesi* markers [29]. Based on the pairwise comparison analysis among genotypes, most isolates were identical by 3 or 4 markers (Fig 2). In samples from the Krueng Sabee and Saree regions, all ten loci had scores considered polymorphic. From the Lhoong and Kuta Cot Glie–Indrapuri regions, nine of ten polymorphic markers were observed; the tenth marker had the same allele size at locus CD13_107.

Multiple identical haplotypes were observed from the Lhoong and Saree regions: three identical haplotypes from the Lhoong population and two pairs of identical haplotypes in Saree. One isolate from Krueng Sabee scored two peaks at locus NC12_4. An isolate from Lhoong resulted in two peaks at marker CD08_61. Further, another isolate from Lhoong resulted in twin peaks at two markers, NC03_2 and NC10_1. The overall index of association amongst isolates from Aceh was *I*_*A*_^*S*^ =  0.008 (p-value =  1.5 ×  10^−1^), which indicated low linkage disequilibrium (LD). All identical haplotypes were from the same population.

### Overall population structure

**Fig 3:**
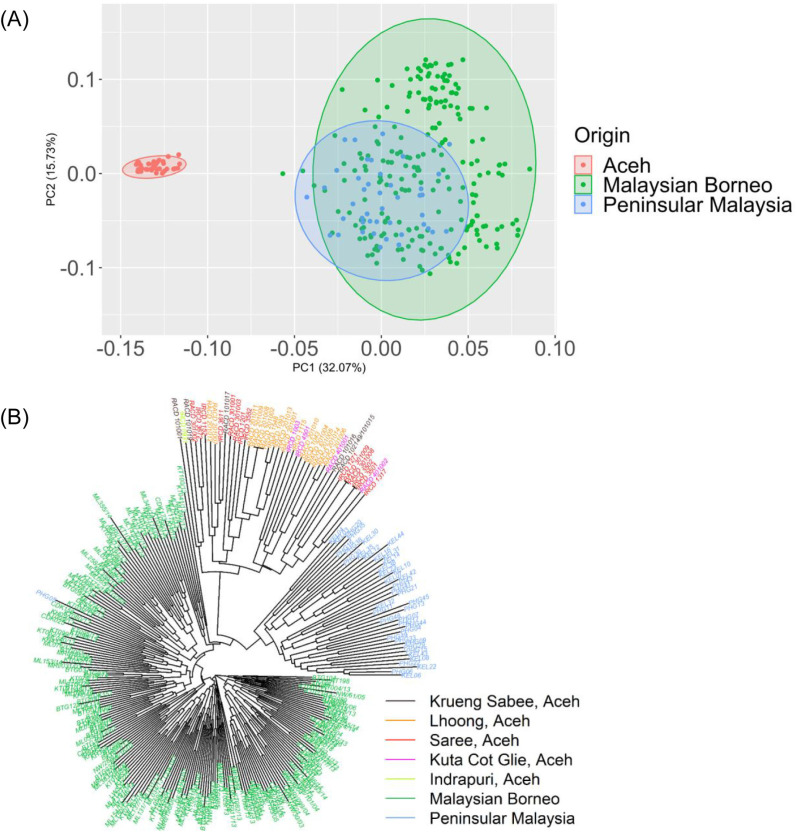
Population structure of *P. knowlesi* from Aceh, Indonesia versus Malaysian Borneo and Peninsular Malaysia. (A) PCoA of *P. knowlesi* samples from Aceh (n = 41), Malaysia Borneo (n = 216), and Peninsular Malaysia (n = 43). (B) Unrooted neighbour-joining tree illustrating the genetic relatedness between *P. knowlesi* from Aceh, Malaysian Borneo, and Peninsular Malaysia.

PCoA and neighbour-joining tree analysis using the *P. knowlesi* sample population compared to *P. knowlesi* samples from Peninsular and Borneo Malaysia resulted in a distinct separation between groups of points (Fig 3). Overall, the results of the PCoA demonstrated a large scatter of points across the plot and no clear group of points (Fig 3a). Similarly, the results of the neighbour-joining tree analysis did not indicate a clear sub-population (Fig 3b). Based on the diagram, isolates from Aceh Province clustered closely together yet were distant from other known *P. knowlesi* populations. Additionally, isolates from Aceh Province clustered more proximally to isolates from Peninsular Malaysia compared to isolates from Malaysian Borneo.

**Fig 4:**
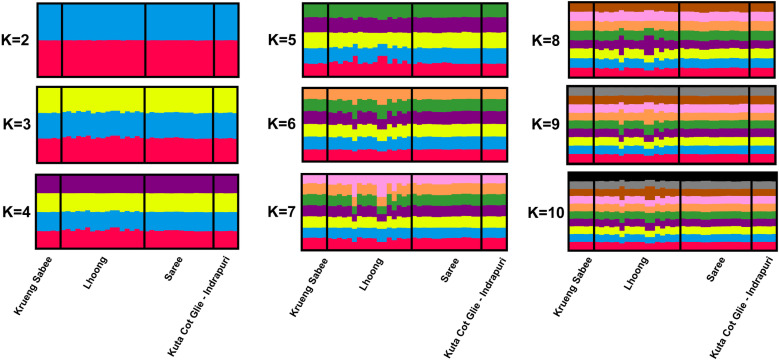
Population structure of *P. knowlesi* in Aceh. Bar plots illustrating the population structure at K =  2–10 in *P. knowlesi* isolates from Aceh. A single vertical bar represents an individual sample, and colors represent the K cluster (sub-population) defined by STRUCTURE. (K1 = red; K2 = blue; K3 = yellow; K4 = purple, K5 = green, K6 = orange, K7 = pink, K8 = brown, K9 = grey, K10 = black).

Analysis using Evano’s test also suggested lack of substructure in the study population. The most probable number of clusters in this population was 2, given that the Δ*K* value was the highest (Δ*K* = 18.9) when K = 2 (S1 Fig, S2 Table). Δ*K* values were all < 1 when K ranged from 3–10. However, assessment of the differentiation between clusters 1 (K1) and 2 (K2) revealed no apparent local substructure in the Aceh Besar and Aceh Jaya populations (F_ST_ =  0.05202, P < 0.005) (Fig 4). Further, based on simulating values of K = 2–10, it appeared unlikely that there was substructure, as there was an equal admixture of both clusters (K1 and K2). It was concluded that there was only one true cluster in the population.

## Discussion

In this genetic surveillance study of *P. knowlesi* conducted over a four-year period in Aceh Province, a near elimination setting, we analyzed 41 cases of *P. knowlesi* infection using microsatellite genotyping of 10 known markers. For samples within a site, nearly all or all loci were polymorphic. Across sites, multiple identical haplotypes were observed, though linkage distribution in the population was low. As reflected in HE, MOI, and F_ST_ values, the parasite population showed low diversity and low complexity. PCoA and neighbour-joining tree analyses provided evidence that *P. knowlesi* strains from Aceh were distinct from those reported in Malaysia, where the large majority of malaria infections over this period were *P. knowlesi*.

Genotyping of *P. knowlesi* with microsatellite markers has previously been performed on other Malaysian *P. knowlesi* populations [29,30]. To our knowledge, this is the first report of genetic diversity and population structure of *P. knowlesi* in Indonesia, specifically. Similar to *P. knowlesi* in Malaysia, we also observed a low index of association and minimal genetic differentiation amongst isolates. Each isolate was relatively geographically close to each other, without natural borders between regions. This phenomenon suggests possible inbreeding of the parasite and/or infrequent introduction of novel *P. knowlesi* genotypes into the population. Thus, a limited number of *P. knowlesi* genotypes spread with low genetic recombination occurrence. Low LD can be reinforced by recombination of *P. knowlesi* in mosquitoes following macaque blood meals containing multiple parasite genotypes [29]. Furthermore, we provide evidence that diversity of isolates was low, and most infections were caused by a single, predominant genotype of *P. knowlesi* with a low MOI number. Low MOIs from high and low endemic areas of *P. knowlesi* infections [30] in addition to populations without structure [31] have previously been reported.

We used the same primers to amplify ten microsatellite markers [29], allowing for comparison between the sites. Significant genetic isolation by geographic location was observed among clusters of isolates from Peninsular Malayasia, Malaysian Borneo, and Aceh Province. The findings show that population of different origins can be distinguished through this method.

There were some limitations of the study. First, while we provide characterization of the genetic diversity of *P. knowlesi* in this area, whole genome sequencing of the study isolates would provide a more robust dataset and the ability to compare *P. knowlesi* isolates to macaque isolates [32]. Second, our samples may not reflect the true population of samples in the study area. It is well established that RDTs and microscopy have limited sensitivity for detecting low-density infections. In passive surveillance of malaria among patients presenting with suspected malaria, low density infections may have been missed by microscopy. *P. knowlesi* has also been reported to cause asymptomatic infection [33] and our study did not include samples detected through active case detection. However, given the low diversity, complexity, and population structure of parasites across sites, it is unlikely that missed samples within sites would have altered these findings. Lastly, given the small number of samples, and limited geographic scope, we were not able to conduct analyses to examine trends over time, and findings cannot be generalized to other areas of the country or outside Indonesia.

Indonesia aims to eliminate the human malaria species by 2030 [34] and control *P. knowlesi* transmission to levels where it is not a significant burden. In 2021, Aceh Besar eliminated malaria, and Aceh Jaya maintained its low-endemicity status. Other progress towards reducing case loads and elimination overall has been made in terms of providing adequate access to malaria case management, strengthening malaria surveillance systems, and reenforcing community engagement and behaviour change communication programs [8,35]. Yet, locally acquired malaria cases continue to occur. Those who engage in forest-related work are at high risk for infection with *P. knowlesi*, as monkeys may be more present in the areas around logging sites and forest plantations [36,37]. The genetic data presented here is a positive indicator that correlates with the epidemiological data, suggesting that the overall low burden of *P. vivax* and *P. falciparum*, along with the low transmission of *P. knowlesi*, highlights that the Aceh region is moving closer to reaching the malaria elimination targets.

## Conclusion

In a near-elimination setting in Indonesia, we found for the first time that *P. knowlesi* strains showed low diversity and low complexity, and were genetically distinct from Malaysian strains, suggesting highly localized transmission and limited connectivity to Malaysia. Ongoing genetic surveillance of *P. knowlesi* in Indonesia can inform tracking and planning of malaria control and elimination efforts.

## Supporting information

S1 Fig Δ*K* value at each assumed *K*-value in STRUCTURE.
The most probable cluster value is n =  2 based on the highest delta value from 20 replicates testing cluster value n =  2 – 10. Data must be used with population structure to test true admixture in sample group.
(TIF)

S1 Table Allele scores of 10 microsatellite loci on *P. knowlesi* from Aceh, Indonesia.
A summary of genotyping results of 41 *P. knowlesi* infections from Krueng Sabee, Lhoong, Saree, Kuta Cot Glie and Indrapuri. Multiple alleles are shaded in yellow and dominant alleles are shown in bold.
(DOCX)

S2 Table Δ*K* value at each assumed *K-*value in STRUCTURE.
The numeral data to graph delta K from sample group.
(DOCX)
