# Supplementary figures and images for: Characterizing the genetic diversity and population structure of Plasmodium knowlesi in Aceh Province, Indonesia
Source: PLoS One. 2025 Mar 11;20(3):e0318608. doi: 10.1371/journal.pone.0318608 (PMC11896071; doi:10.1371/journal.pone.0318608)

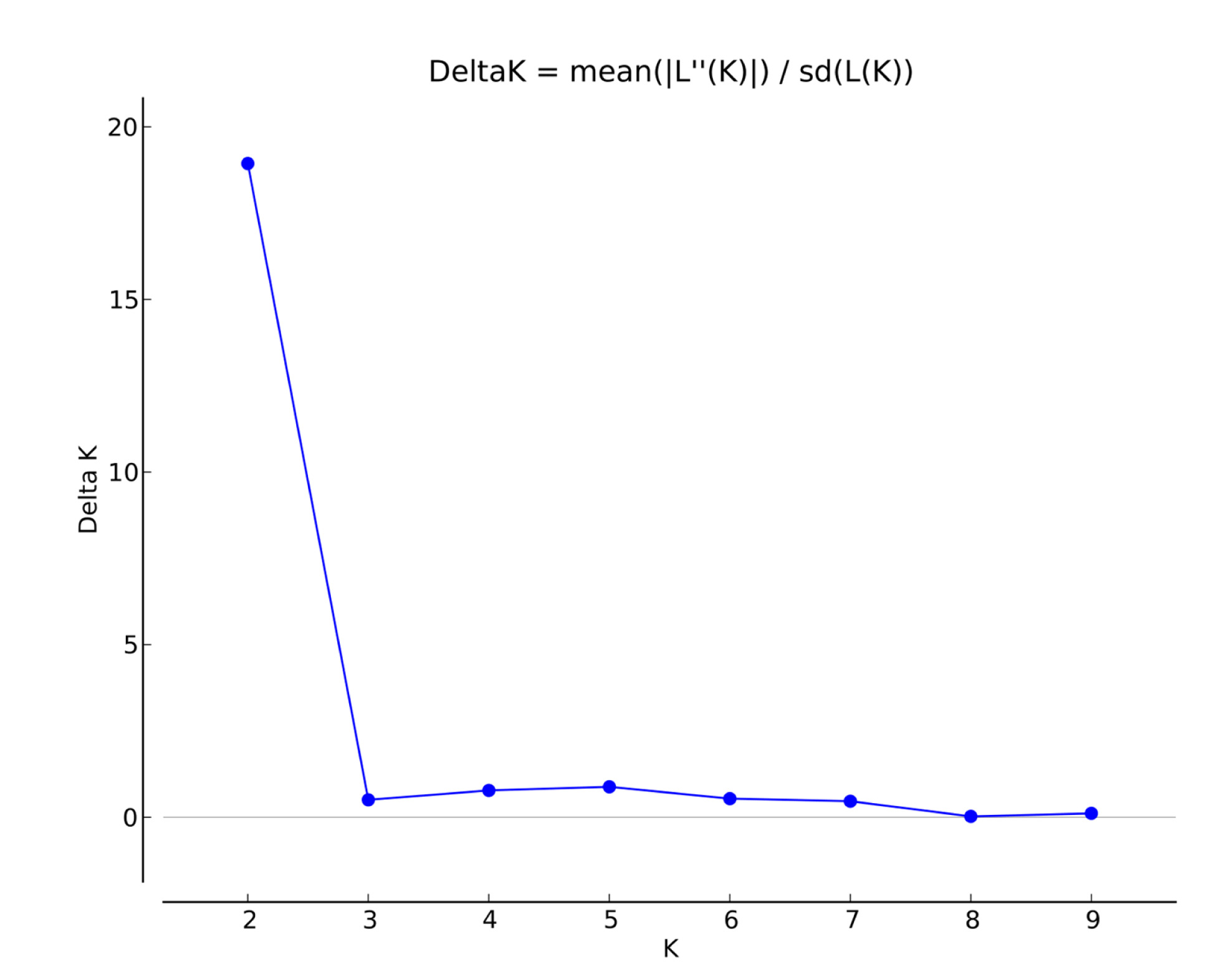

Supplement: S1 Fig — The most probable cluster value is n = 2 based on the highest delta value from 20 replicates testing cluster value n = 2 – 10. Data must be used with population structure to test true admixture in sample group. (TIF) [file pone.0318608.s001.tif]
