# Supplementary material for: Characterizing the genetic diversity and population structure of Plasmodium knowlesi in Aceh Province, Indonesia
Source: PLoS One. 2025 Mar 11;20(3):e0318608. doi: 10.1371/journal.pone.0318608 (PMC11896071; doi:10.1371/journal.pone.0318608)
Supplement: S1 Table — A summary of genotyping results of 41 P. knowlesi infections from Krueng Sabee, Lhoong, Saree, Kuta Cot Glie and Indrapuri. Multiple alleles are shaded in yellow and dominant alleles are shown in bold. (DOCX) [file pone.0318608.s002.docx]

**Supporting information**

**S1 Table. Allele scores of 10 microsatellite loci on *P. knowlesi* from Aceh, Indonesia.**

| **No.** | **Sample ID** | **Origin** | **NC03_2** | **CD11_157** | **NC12_4** | **NC12_2** | **CD13_61** | **CD08_61** | **NC10_1** | **CD13_107** | **CD05_06** | **NC09_1** |
| --- | --- | --- | --- | --- | --- | --- | --- | --- | --- | --- | --- | --- |
| 1 | RACD 101001 | Krueng Sabee | **138** | **252** | 231 / **234** | **342** | **177** | **225** | **270** | **180** | **249** | **282** |
| 2 | RACD 102149/101015 | Krueng Sabee | **147** | **252** | **234** | **327** | **174** | **231** | **276** | **189** | **261** | **282** |
| 3 | RACD 101016 | Krueng Sabee | **147** | **249** | **234** | **324** | **174** | **228** | **279** | **189** | **312** | **282** |
| 4 | RACD 101017 | Krueng Sabee | **138** | **240** | **234** | **345** | **177** | **216** | **276** | **192** | **249** | **285** |
| 5 | RACD 101019 | Krueng Sabee | **138** | **249** | **228** | **324** | **177** | **231** | **270** | **189** | **366** | **282** |
| 6 | IRCD 1043 | Lhoong | **138** | **243** | **231** | **324** | **174** | **216** | **270** | **189** | **246** | **303** |
| 7 | IRCD 1093 | Lhoong | **141** | **243** | **234** | **327** | **174** | **210** | **267** | **189** | **249** | **285** |
| 8 | IRCD 1115 | Lhoong | **147** | **246** | **234** | **327** | **171** | **231** | **294** | **189** | **246** | **285** |
| 9 | IRCD 1548 | Lhoong | **138** / 141 | **243** | **234** | **327** | **174** | **210** | **267** / 270 | **189** | **249** | **285** |
| 10 | RACD 201001 | Lhoong | **138** | **243** | **234** | **318** | **174** | **216** / 231 | **276** | **189** | **255** | **285** |
| 11 | RACD 201002 | Lhoong | **138** | **246** | **234** | **321** | **174** | **216** | **270** | **189** | **249** | **285** |
| 12 | RACD 201004 | Lhoong | **138** | **249** | **234** | **393** | **180** | **216** | **267** | **189** | **252** | **282** |
| 13 | RACD 201005 | Lhoong | **138** | **249** | **234** | **315** | **174** | **216** | **270** | **189** | **246** | **282** |
| 14 | RACD 201006 | Lhoong | **138** | **249** | **243** | **327** | **174** | **228** | **294** | **189** | **246** | **282** |
| 15 | RACD 201007 | Lhoong | **138** | **249** | **234** | **327** | **177** | **216** | **276** | **189** | **249** | **285** |
| 16 | RACD 201008 | Lhoong | **138** | **246** | **234** | **321** | **174** | **216** | **270** | **189** | **249** | **285** |
| 17 | RACD 201009 | Lhoong | **138** | **246** | **234** | **321** | **174** | **216** | **270** | **189** | **249** | **285** |
| 18 | RACD 201010 | Lhoong | **138** | **246** | **231** | **315** | **177** | **216** | **279** | **189** | **303** | **303** |
| 19 | RACD 201011 | Lhoong | **138** | **255** | **234** | **324** | **174** | **216** | **270** | **189** | **249** | **285** |
| 20 | RACD 201012 | Lhoong | **141** | **249** | **243** | **324** | **177** | **216** | **276** | **189** | **246** | **285** |
| 21 | RACD 201013 | Lhoong | **138** | **243** | **234** | **321** | **174** | **210** | **276** | **189** | **246** | **285** |
| 22 | RACD 201014 | Lhoong | **138** | **249** | **234** | **327** | **174** | **228** | **294** | **189** | **246** | **282** |
| 23 | IRCD 1152 | Saree | **141** | **249** | **234** | **342** | **177** | **225** | **276** | **180** | **276** | **285** |
| 24 | IRCD 1201 | Saree | **138** | **249** | **231** | **324** | **174** | **216** | **279** | **189** | **249** | **285** |
| 25 | IRCD 1271 | Saree | **138** | **249** | **234** | **321** | **183** | **225** | **267** | **189** | **246** | **282** |
| 26 | IRCD 1317 | Saree | **141** | **246** | **234** | **327** | **174** | **225** | **288** | **189** | **267** | **282** |
| 27 | IRCD 3501 | Saree | **141** | **246** | **234** | **399** | **174** | **228** | **267** | **189** | **249** | **282** |
| 28 | IRCD 3502 | Saree | **141** | **246** | **234** | **399** | **174** | **228** | **267** | **189** | **249** | **282** |
| 29 | IRCD 3552 | Saree | **138** | **243** | **234** | **315** | **174** | **231** | **282** | **189** | **249** | **285** |
| 30 | IRCD 3611 | Saree | **138** | **240** | **234** | **318** | **177** | **216** | **285** | **189** | **249** | **285** |
| 31 | IRCD 3672 | Saree | **138** | **249** | **231** | **318** | **177** | **231** | **288** | **189** | **249** | **282** |
| 32 | RACD 301002 | Saree | **138** | **249** | **234** | **324** | **177** | **231** | **288** | **189** | **246** | **282** |
| 33 | RACD 301001 | Saree | **138** | **246** | **228** | **354** | **174** | **225** | **267** | **192** | **297** | **285** |
| 34 | RACD 301003 | Saree | **141** | **249** | **237** | **315** | **174** | **225** | **279** | **189** | **279** | **285** |
| 35 | RACD 301008 | Saree | **138** | **252** | **234** | **345** | **174** | **225** | **288** | **189** | **315** | **282** |
| 36 | RACD 301009 | Saree | **138** | **252** | **234** | **345** | **174** | **225** | **288** | **189** | **315** | **282** |
| 37 | IRCD 1003 | Kuta Cot Glie | **138** | **246** | **234** | **345** | **177** | **228** | **282** | **189** | **246** | **285** |
| 38 | IRCD 4501 | Kuta Cot Glie | **138** | **252** | **231** | **333** | **174** | **216** | **282** | **189** | **246** | **282** |
| 39 | RACD 401001 | Kuta Cot Glie | **147** | **249** | **234** | **324** | **174** | **225** | **267** | **189** | **246** | **291** |
| 40 | RACD 401002 | Kuta Cot Glie | **141** | **246** | **234** | **318** | **174** | **225** | **276** | **189** | **246** | **282** |
| 41 | IRCD 0931 | Indrapuri | **138** | **255** | **237** | **324** | **177** | **225** | **288** | **189** | **249** | **291** |

A summary of genotyping results of 41 *P. knowlesi* infections from Krueng Sabee, Lhoong, Saree, Kuta Cot Glie and Indrapuri. Multiple alleles are shaded in yellow and dominant alleles are shown in bold.
