# Supplementary material for: Characterizing the genetic diversity and population structure of Plasmodium knowlesi in Aceh Province, Indonesia
Source: PLoS One. 2025 Mar 11;20(3):e0318608. doi: 10.1371/journal.pone.0318608 (PMC11896071; doi:10.1371/journal.pone.0318608)
Supplement: S2 Table — The numeral data to graph delta K from sample group. (DOCX) [file pone.0318608.s003.docx]

**Supporting information**

**S2 Table.** $\boldsymbol{\Delta}$***K* value at each assumed *K-*value in STRUCTURE.**

| **K** | **Reps** | **Mean LnP(K)** | **Stdev LnP(K)** | **Ln’(K)** | **\|Ln”(K)\|** | **Delta K** |
| --- | --- | --- | --- | --- | --- | --- |
| 1 | 20 | -565.86 | 0.88876 | - | - | -- |
| 2 | 20 | -566.025 | 1.627033 | -0.165 | 30.815 | 18.939381 |
| 3 | 20 | -597.005 | 41.025069 | -30.98 | 20.76 | 0.506032 |
| 4 | 20 | -607.225 | 50.661808 | -10.22 | 39.41 | 0.777904 |
| 5 | 20 | -656.855 | 64.212849 | -49.63 | 56.62 | 0.881755 |
| 6 | 20 | -649.865 | 90.955653 | 6.99 | 49.105 | 0.539878 |
| 7 | 20 | -691.98 | 115.20723 | -42.115 | 53.53 | 0.464641 |
| 8 | 20 | -680.565 | 123.072509 | 11.415 | 3.05 | 0.024782 |
| 9 | 20 | -672.2 | 128.443397 | 8.365 | 14.555 | 0.113318 |
| 10 | 20 | -678.39 | 136.908955 | -6.19 | - | - |

The numeral data to graph delta K from sample group.
